# Protective effect of *Lavandula angustifolia* essential oil inhalation on neuromodulators regulating the sleep/wake cycle in rats with total sleep deprivation

**DOI:** 10.22038/ijbms.2024.78085.16880

**Published:** 2025

**Authors:** Arzu Yalcin, Mustafa Saygin, Ozlem Ozmen, Rahime Aslankoc, Önder Özturk, Hasan Aslancan, Oguzhan Kavrik

**Affiliations:** 1 Department of Physiology, Faculty of Medicine, Suleyman Demirel University, Isparta, Turkey; 2 Department of Pathology, Burdur Mehmet Akif Ersoy University Faculty of Veterinary Medicine, Burdur, Turkey; 3 Department of Chest Diseases, Faculty of Medicine, Suleyman Demirel University, Isparta, Turkey; 4 Fruit Research Institute, Eğirdir/Isparta, Turkey

**Keywords:** Basal forebrain, Deprivation, Lavender oil, Locus coeruleus, Neural protection, Preoptic area, Sleep

## Abstract

**Objective(s):**

This study aimed to investigate the potential effects of different doses of *Lavender angustifolia* essential oil (Lavender EO) administered by inhalation on sleep latency and neuromodulators regulating the sleep/wake cycle in rats with total sleep deprivation (TSD).

**Materials and Methods:**

Forty-eight male Sprague-Dawley rats were divided into five groups: Control, Alprazolam (ALP, 0.25 mg/kg given intraperitoneally), L1 (Lavender EO, 0.3 ml given by inhalation), L2 (Lavender EO, 0.5 ml given by inhalation), and L3 (Lavender EO, 1 ml given by inhalation); TSD was applied to all groups. Rats in SD groups were kept on a platform surrounded by water for 18 hr for 20 days, and for the remaining time, the animals were exposed to Lavender EO for 1 hr (11:00–12:00) and then were kept in their home cage for 5 hr (12:00-17:00). Their brain and brainstem were removed for histopathological and immunohistochemical analyses (c-Fos, ChAT, GAD, and ADRB2 expression) in the locus coeruleus (LC), basal forebrain (BF), and preoptic area (PO).

**Results:**

The groups ranked by the severity of edema, hyperemia, and neurodegeneration in LC, BF, and PO areas were control, L3, L1, L2, and ALP. c-Fos expression significantly decreased in all brain regions in all groups except the L1 group. ChAT and GAD expressions increased dramatically in all brain regions. ADRB2 significantly increased in LC in ALP and L2 groups; in the PO area in ALP, L1, and L2 groups; and in BF in all groups.

**Conclusion:**

Lavender EO treatment ameliorated c-Fos, ChAT, GAD, and ADRB2 expression, similar to the effect of ALP.

## Introduction

During sleep, various biological and physiological processes occur, including regulating blood pressure, endocrine and immune system functions, cellular repair, body temperature control, and restoration of cognitive functions (1). Therefore, sleep plays a critical role in maintaining and protecting physical and mental health throughout an individual’s life (2). 

In developed societies, most people experience inadequate sleep, disruptions in circadian rhythms, and increased fatigue. This can be attributed to electronic devices changing melatonin secretion, intense work tempo, shift work, and other lifestyle-related issues such as age-related sleep deprivation (3). According to several economic analyses, insufficient sleep causes an increased prevalence of diseases, loss of work performance, and an elevated risk of accidents (4). Sleep deprivation (SD) also affects psychomotor functions and working memory, causing the impairment of basic cognitive functions such as attention and learning (5). At the same time, SD causes metabolic dysfunctions such as obesity, diabetes, cardiovascular diseases, and immune system dysfunction (6).

In recent years, there has been an increasing number of publications on memory consolidation during sleep, post-sleep restoration, and deficiencies following sleep disturbance. The hallmark of the sleep/wake cycle is the systematic change in the activity of neuromodulatory neurons (7). Noradrenaline (NA) and gamma-aminobutyric acid (GABA) are among the major neurotransmitters regulating rapid eye movement (REM) sleep. Changes in NA and GABA levels in different brain regions will likely maintain or disrupt the sleep/wake cycle (8). The significance of the cholinergic system in both formation and maintenance of REM sleep is widely recognized (9). Earlier research has identified a correlation between the cholinergic system, body temperature, and circadian rhythm. Additionally, it has been shown that the cholinergic system is directly involved in regulating the sleep cycle, along with cognitive processes, such as learning-memory and attention (10, 11). Memory restoration does not solely occur spontaneously during sleep; external stimuli such as odors can also reactivate specific memories. In this context, previous research has shown that memories of object locations could be reactivated during sleep by re-exposing subjects to odor cues associated with previously learned object locations (12).

*Lavandula angustifolia* is a leafy, fragrant, shrub-shaped perennial plant that is native to the Mediterranean region and is a prominent member of the Lamiaceae family (13). *L. angustifolia *essential oil (Lavender EO) has long been used in traditional medicine to cure insomnia, depression, and nervous disorders (14). Nowadays, Lavender EO is recognized for its various therapeutic effects, such as antibacterial (15), anti-inflammatory (16), analgesic (17), antistress, anxiolytic, and sedative effects (18). It has also been reported that it accelerates the onset of sleep and improves sleep quality, but information on this subject is rather limited.

Due to developing technology and intense work pace, many people experience chronic sleep deprivation and sleep disorders (3). Medications used in modern medicine facilitate sleep and prolong sleep duration but do not improve sleep quality. Therefore, there is a need for a treatment that is safe and devoid of adverse effects. Aromatic essential oils, considered risk-free with a few side effects, may have promising potential as a therapeutic agent in treating sleep disorders (19). The current study aims to determine the healing effect of Lavender EO inhalation on neuromodulators regulating the sleep/wake cycle in rats with sleep disorders and to develop an alternative treatment model.

## Materials and Methods

***Animals***

The study was approved by the Animal Experiments Local Ethics Committee of Süleyman Demirel University (Approval No: 23.09.2021/01). The experiment was carried out in accordance with the guidelines on animal care and experiments of the European Communities Council Directive (86/609/EEC). Animals were obtained from SDÜ Animal Production Laboratory and Experimental Research Laboratory and kept in SDÜ Animal Production Laboratory and Experimental Research Laboratory during the experiment.

***Study groups***

**Figure 1:**
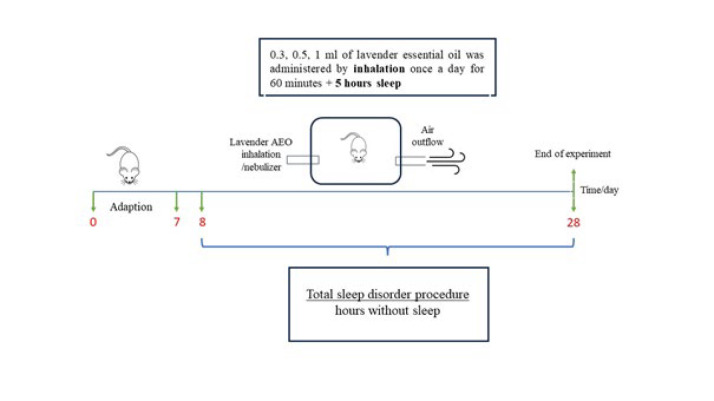
Experimental procedure testing the effects of lavender AEO inhalation in rats

The experimental procedure design is shown in Figure 1. Our study used forty-eight male Sprague-Dawley rats (12-14 weeks old and weighing 250-300 g). All animals were fed a standard chow, with food and water present *ad libitum*, and kept in a controlled environment (temperature 21-23 ^°^C, 12-hour light/dark cycle, and humidity 55-60%). All animals were subjected to total sleep deprivation (TSD). After 1-week adaptation to laboratory conditions, the rats in all groups were placed on multiple small platforms as described in the experimental procedure, while rats in the control group were placed on a grid under the same conditions. Lavender EO inhalation application was performed between 11:00 and 12:00. During the experiment, the animals’ weight was checked on a weekly basis. The experimental groups were created as follows:

Control group (Control, n=8): The animals underwent SD, received 0.5 ml of saline inhalation for 60 min, and were placed on a grid under the same conditions as the other groups.

Alprazolam group (ALP, n=10): The animals underwent SD and received 0.25 mg/kg of Alprazolam dissolved in saline (20)(ALP, Pfizer, Xanax, USA) by intraperitoneal injection once a day. 0.5 ml of saline inhalation for 60 min was administered once a day.

Lavender EO-1 group (L1, n=10): The animals underwent SD and received 0.3 ml of Lavender EO inhalation once a day for 60 min. 

Lavender EO-2 group (L2, n=10): The animals underwent SD and received 0.5 ml of Lavender EO inhalation once a day for 60 min. 

Lavender EO-3 group (L3, n=10): The animals underwent SD and received 1 ml of Lavender EO inhalation once a day for 60 min.

After performing the experimental procedure, the animals were sacrificed under ketamine (90 mg/kg) and xylazine (10 mg/kg) anesthesia. To evaluate the expression of c-Fos, ChAT, GAD, and ADRB2 in the locus coeruleus (LC), preoptic area (PO), and basal forebrain (BF), the brain and brainstem were gently dissected and placed in 10% formaldehyde for histopathological and immunohistochemical examinations.

***Total sleep deprivation procedure***

The TSD model was designed with reference to previous studies (21, 22). SD was performed in a plexiglass water tank of 145 cm in length, 44 cm in width, and 45 cm in height. Twelve narrow circular platforms (6.5 cm in diameter) were placed inside the tank filled with water to 1 cm of their upper surface. Ten rats were placed in each cage, leaving two platforms empty. The rats were able to move freely and jump from one platform to another. Animals experiencing muscle atony during the paradoxical sleep awakened upon falling from the platform into the water, disrupting their sleep cycle. The housing conditions of the control group were similar. The animals were allowed to sleep on a grid floor expanded from the cage, even though the rats’ tails might have touched the water below. After one week of adaptation, the rats in SD groups were kept on the platform for 18 hr for 20 days. During the remaining time, the animals were exposed to Lavender EO (1 hr, 11:00-12:00) and then were allowed to stay in their home cage (5 hr, 12:00-17:00). Environmental conditions were kept constant throughout the experiment (temperature 21-23 °C, 12-hour light/dark cycle). The rats were provided unlimited access to food and water.

***Isolation of essential oils***

The Lavender EO (*Lavandula angustifolia)* investigated in this study was purchased from a local company (Health and Sleep R&D, Turkey). EO was extracted using the hydrodistillation method and used as pure oil without further modification. SDÜ Natural Products Application and Research Center analyzed the oil content (Report number: 2108036, Report date: 19.08.2021).

***Instrumentation and equipment details***

Lavender EO analysis was performed by a gas chromatograph - a mass spectrometry detector (GC-MSD). Determination of terpenoid compounds was performed by Thermo Scientific TRACE 1300 gas chromatography combined with Thermo Scientific ISQ 8000 mass spectrometer detector and Thermo Scientific RSH Triplus autosampler (Thermo Fisher Scientific Inc. Waltham, MA, USA). Chromatographic evaluations were performed using Xcalibur software. TRACE TR-5MS capillary GC column (5% phenyl 95% dimethyl polysiloxane, 30 m×0.25 mm, 0.25 μm film thickness) (Thermo Fisher Scientific Inc. Waltham, MA, USA) was used as the analytical column for chromatographic separation. The oil sample was diluted with acetone at a ratio of 1:500 in a 2 ml vial and injected into the GC-MSD system. The entire analysis took 65 min. The injections were performed as follows: inlet temperature 250 ^°^C, injection volume 2 μl, split ratio 1:20. Helium was used as the carrier gas with a 1.5 ml/min flow. The oven temperature was initially held at 30 ^°^C for 5 min, then increased by 5 ^°^C/min to 280 ^°^C and held at this level for 10 min. The detector temperature was 230 ^°^C. 

The compounds were identified by comparison based on spectra reported on Willey 1n.l and NIST 0.5 databases (National Institute of Standards and Technology). For each identified compound, basic, molecular, and qualifying ions were selected as part of the identification process.

***Lavender essential oil application procedure***

In this study, an airtight cylindrical plastic tank (height: 28 cm, width: 40 cm, length: 60 cm) was used for Lavender EO inhalation. 2 holes (3 cm in diameter) for the inlet and outlet of air flow were drilled in the tank. An inhalation device (OMRON NE-C28P-E class II type BF Compressor Nebulizer, Kyoto, Japan) was connected to one of the holes to blow Lavender EO into the tank. The other hole was used for the air outflow. Lavender EO was applied to L1, L2, and L3 groups (0.3 ml, 0.5 ml, and 1 ml, respectively) for 1 hr. The positive control group received Alprazolam (0.25 mg/kg) intraperitoneally.

***Histopathological method***

Brain samples were collected immediately after decapitation during autopsy and fixed in 10% buffered formaldehyde solution for two days. Subsequently, the tissues were trimmed to include the BF, PO area, and locus coeruleus and placed in tracking cassettes. After one more day in formaldehyde, the cassettes were placed in a fully automated tissue processor (Leica ASP300S; Leica Microsystem, Nussloch, Germany) for routine tissue tracking. The tissues kept in cassettes were passed through a series of ascending alcohols (70% to 100%) overnight to remove the water from the tissues. The fat was then removed from the tissues by passing through two xylols, followed by hot paraffin to fill the tissue cavities. The tissues were left in the processor overnight, and the next morning, they were embedded in paraffin to obtain paraffin blocks. 

After cooling for 4-5 hr, 5-µm thick serial sections were taken from each block using a Leica 2155 rotary microtome for both histopathological and immunohistochemical examinations. The sections taken from normal slides were stained with a routine hematoxylin-eosin (HE) stain. For this purpose, the brain sections mounted on slides were initially dehydrated in an oven at 60 ^°^C for 2 hr and subsequently paraffinized by passing through 3 different xylol series for 30 min. Then, the tissues were rehydrated by passing through a series of descending alcohols (100%, 96%, 90%, 80%, and 70% alcohol, respectively). Then, H&E staining was performed using Harris hematoxylin for 15 min and then eosin for 3 min. After staining, the tissues were completely dehydrated by passing through a series of ascending alcohols (70%, 80%, 90%, 96%, and 100% alcohol, respectively). Entellan was applied to the tissues, which had been polished with xylene and covered with coverslips, before being examined under a microscope.

The following parameters were evaluated to score brain injury: perivascular edema, perivascular hemorrhages, congestion, neuronal damage, and hyperacidophilia. A semi-quantitative analysis was performed, with values of 0 (no lesion observed), 1 (slight lesions), or 2 (marked lesions). 

***Immunohistochemical method***

Immunohistochemical examinations were conducted on the sections prepared on polylysine-coated slides and stained using the streptavidin-biotin-peroxidase method. Polyclonal Anti-Choline Acetyltransferase/ChAT Antibody Picoband (Catalog No: A01192-5 (Boster Bio, CA, USA), GAD Polyclonal antibody (bs-0400R-TR) (Bioss Antibodies Inc., MA, USA), ADRB2 Polyclonal Antibody (bs-0947R-TR) (Bioss Antibodies Inc., MA, USA), and c-Fos (Ser362) (bs-12910R-HRP)(Bioss Antibodies Inc.MA, USA) primary antibodies were used for immunohistochemical analysis. All primary antibodies were used at 1:100 dilution; the sections were deparaffinized and rehydrated in xylol and graded alcohols, as described above. The sections were washed with water for 10 min, then soaked in hydrogen peroxide (3% in methanol) for 20 min to remove endogenous peroxidase activity within the tissues. Afterward, they were boiled two times for 5 min in a citrate buffer solution.

The tissues were washed two times with phosphate-buffered saline (PBS) for 10 min and subsequently incubated in a normal medium. After this step, excess serum was removed without washing, and the samples were incubated with primary antibody overnight at room temperature. All the sections were then washed with PBS in a similar way and period of time, followed by 30 min of streptavidin instillation, and then washed twice with PBS for 10 min. Subsequently, the tissues were incubated with biotin serum for 30 min. The sections were then washed in the same way and for the same period of time. Afterward, staining was performed with freshly prepared DAB (3.3 diaminobenzidine) chromogen. BioVision’s ready-to-use IHC/ICC kit (Biotin Free), One-step HRP Polymer anti-Mouse, and Rat and Rabbit IgG with DAB (Catalog K405-50) were used as the secondary antibody. Harris hematoxylin was used for counterstaining. After all the procedures, the slides were covered with coverslips, dehydrated, and examined under a light microscope. No primary antibody was added to the sections for negative controls, and the staining was completed using a similar method.

To determine the percentage of immunostained cells for each marker, 20 cells from each group were counted in 20 different areas in every section at x40 magnification. Each sample’s percentage of immunostained cells was calculated, and statistical analysis was conducted. Morphometric analysis was performed using the Database Manual CellSens Life Science Imaging Software (Olympus Co., Tokyo, Japan).

***Statistical analysis***

Statistical evaluation was performed using SPSS software version 22.0 for Windows (SPSS Inc., Chicago, IL, USA). The homogeneity of the data was tested using the Shapiro-Wilk and Kolmogorov-Smirnov tests. Statistical analysis of the significant differences between the means of normally distributed data was performed by one-way analysis of variance (ANOVA). Tukey *post hoc* test was used to determine significant differences between the groups’ variables. Data were presented as mean±standard deviation (SD). *P*-values <0.05 were considered statistically significant.

**Figure 2:**
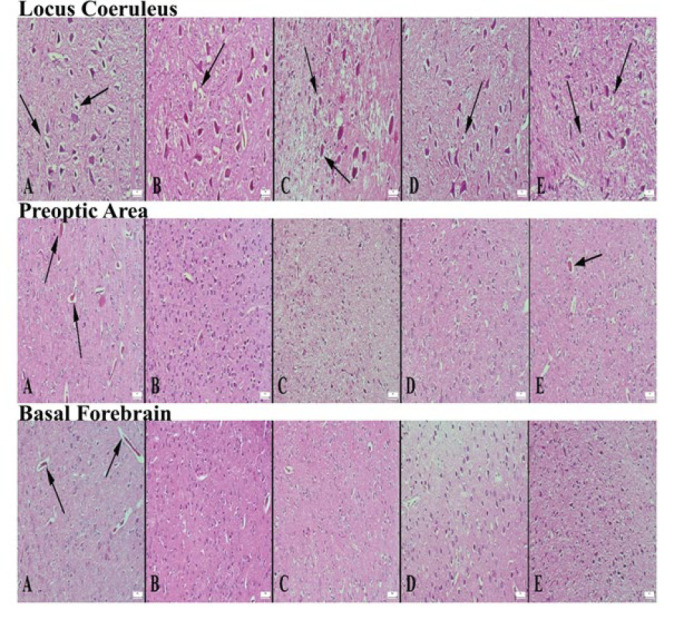
Microscopic appearance of locus coeruleus according to groups of rats (A) Significant degenerative changes in neurons (arrows) in the control group, (B) Significant decrease in degenerative cells (arrow) in the alprazolam group, (C) Reduced degenerative neurons in L1 group compared to control (arrows), (D) markedly reduced degenerative neurons in L2 group (arrow), (E) appearance of mildly reduced degenerative neurons in L3 group compared to control, HE, Bars=50 µm. Microscopic appearance of the preoptic area of the groups (A) marked hyperemia in the vessels in the control group (arrows), (B) markedly decreased pathological findings in the Alprazolam group, (C) decreased findings in the L1 group, (D) similar appearance to Alprazolam in the L2 group, (E) appearance of reduced pathological findings in the L3 group compared to the control, HE, Bars=50 µm. Microscopic appearance of the basal forebrain areas of the groups (A) marked hyperemia in the vessels in the control group (arrows), (B) markedly decreased pathological findings in the Alprazolam group, (C) decreased findings in the L1 group compared to the control group, (D) markedly decreased appearance of pathological findings in the L2 group, (E) appearance of decreased pathological findings in the L3 group compared to the control group, HE, Bars=50µm

**Figure 3:**
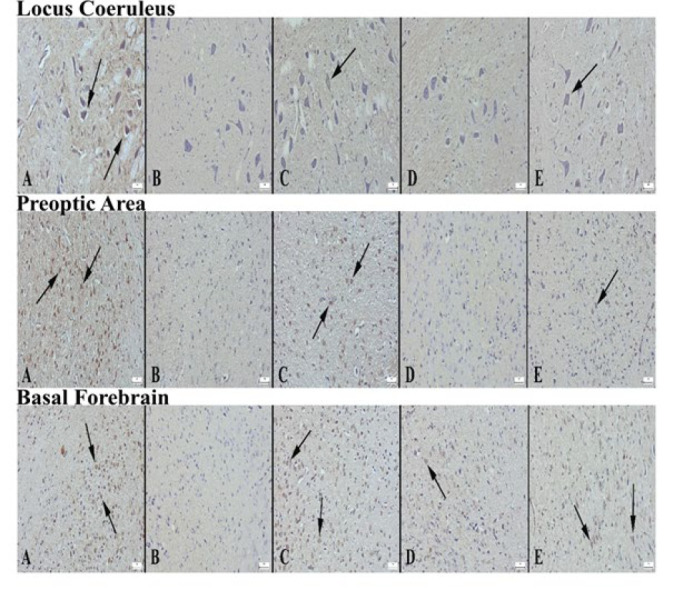
Appearance of c-fos expression in locus coeruleus areas of the groups of rats (A) Significantly increased expression in neurons in control group (arrows), (B) Negative expression in Alprazolam group, (C) Decreased expression in L1 group compared to control group (arrow), (D) Negative expression in L2 group, (E) Decreased expression in L3 group compared to control, Streptavidin biotin peroxidase method, Bars=50 µm. c-fos expression in the preoptic area of the groups (A) Severe expression in neurons in the control group (arrows), (B) Negative expression in the alprazolam group, (C) Significantly decreased expression in the L1 group compared to the control group (arrow), (D) Negative expression in the L2 group, (E) Decreased expression in neurons in the L3 group compared to the control group (arrow), Streptavidin biotin peroxidase method, Bars=50 µm. Appearance of c-fos expression in the basal forebrain regions of the groups (A) Very strong expression in a large number of neurons in the control group (arrows), (B) Negative expression in the alprazolam group, (C) Significantly decreased expression in neurons in the L1 group compared to the control group (arrows), (D) Mild expression in few neurons in L2 group (arrow), (E) Mild expression in fewer neurons in L3 group compared to control group (arrows), Streptavidin biotin peroxidase method, Bars=50 µm

**Figure 4:**
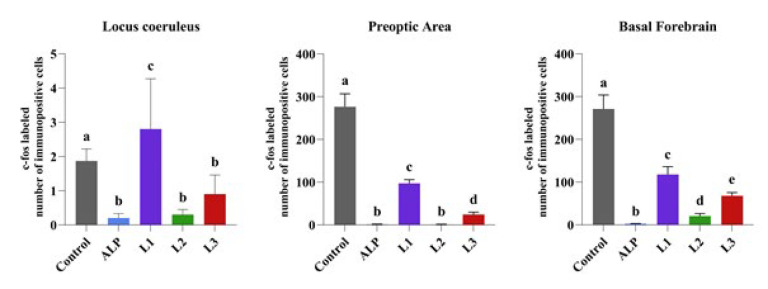
Comparison between groups of rats was evaluated using the Duncan test

Groups with the same superscript in the same column are statistically similar, and the differences between groups with different symbols indicate statistical significance (*P<*0.05). Data are presented as mean and standard deviation

**Figure 5:**
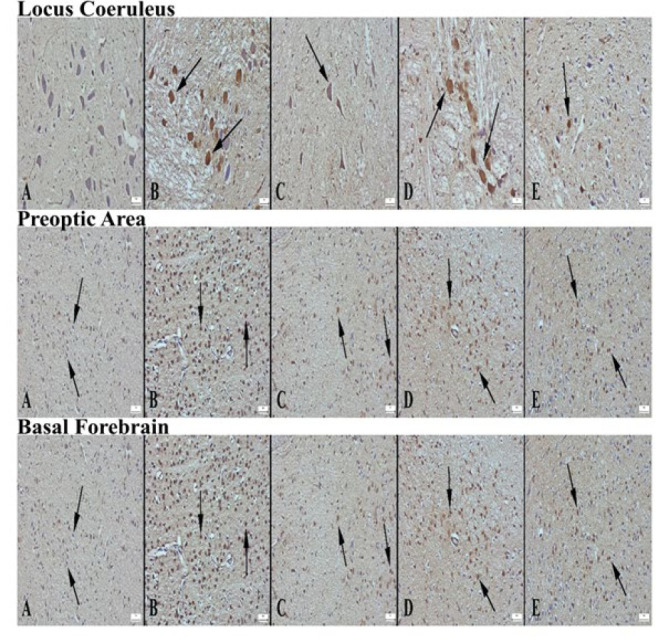
Appearance of ChAT expression in locus coeruleus area of the groups of rats (A) Decreased expression in the control group, (B) Significantly increased expression in the alprazolam group (arrows), (C) Increased expression in L1 neurons compared to control group (arrow), (D) markedly increased expression in L2 group (arrows), (E) increased expression in L3 group compared to control, Streptavidin biotin peroxidase method, Bars=50µm. Appearance of ChAT expression in preoptic area according to groups (A) Decreased expression in neurons in the control group (arrows), (B) Significantly increased expression in neurons in the alprazolam group (arrows), (C) Increased expression in L1 neurons compared to control group (arrows), (D) markedly increased expression in L2 group (arrows), (E) increased expression in L3 group compared to control, Streptavidin biotin peroxidase method, Bars=50 µm. Appearance of ChAT expression in the basal forebrain area of the groups (A) Decreased expression in the control group, (B) Significantly increased expression in the alprazolam group (arrows), (C) Increased expression in L1 neurons compared to control group (arrow), (D) markedly increased expression in L2 group (arrows), (E) increased expression in L3 group compared to control, Streptavidin biotin peroxidase method, Bars=50 µm

**Figure 6:**
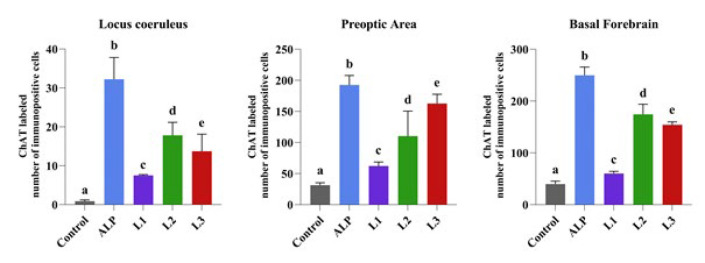
Comparison between groups of rats was evaluated using the Duncan test

Groups with the same superscript in the same column are statistically similar, and the differences between groups with different symbols indicate statistical significance (*P<*0.05). Data are presented as mean and standard deviation

**Figure 7:**
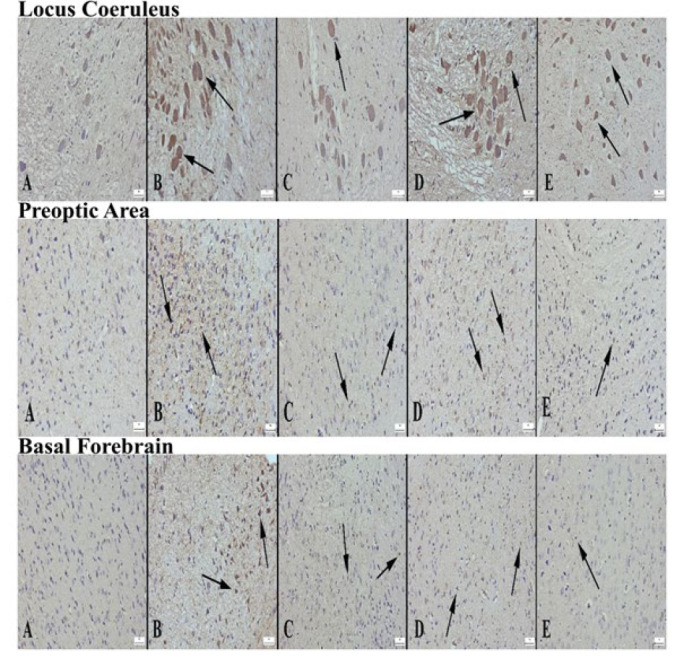
Appearance of GAD expression in locus coeruleus area of the groups of rats (A) Decreased expression in the control group, (B) Significantly increased expression in the alprazolam group (arrows), (C) Increased expression in L1 neurons compared to control group (arrow), (D) Significantly increased expression in L2 group (arrows), (E) Increased expression in L3 group neurons compared to control, Streptavidin biotin peroxidase method, Bars=50 µm. Appearance of GAD expression in preoptic area according to groups (A) decreased expression in neurons in the control group, (B) Significantly increased expression in neurons in the alprazolam group (arrows), (C) Increased expression in neurons of L1 group compared to control group (arrows), (D) markedly increased expression in L2 group (arrows), (E) increased expression in L3 group compared to control, Streptavidin biotin peroxidase method, Bars=50 µm. Appearance of GAD expression in the basal forebrain area of the groups (A) Decreased expression in the control group, (B) Significantly increased expression in the alprazolam group (arrows), (C) Increased expression in L1 neurons compared to control group (arrow), (D) markedly increased expression in L2 group (arrows), (E) increased expression in L3 group compared to control, Streptavidin biotin peroxidase method, Bars=50 µm

**Figure 8:**
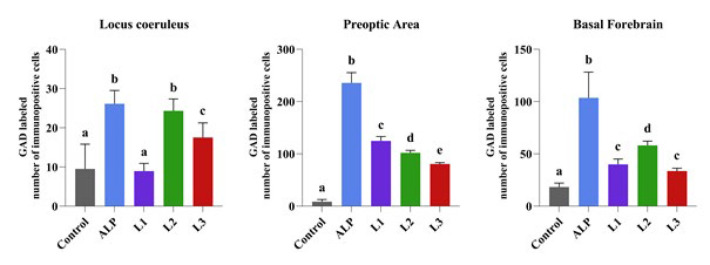
Comparison between groups of rats was evaluated using the Duncan test

Groups with the same superscript in the same column are statistically similar, and the differences between groups with different symbols indicate statistical significance (*P<*0.05). Data are presented as mean and standard deviation

**Figure 9:**
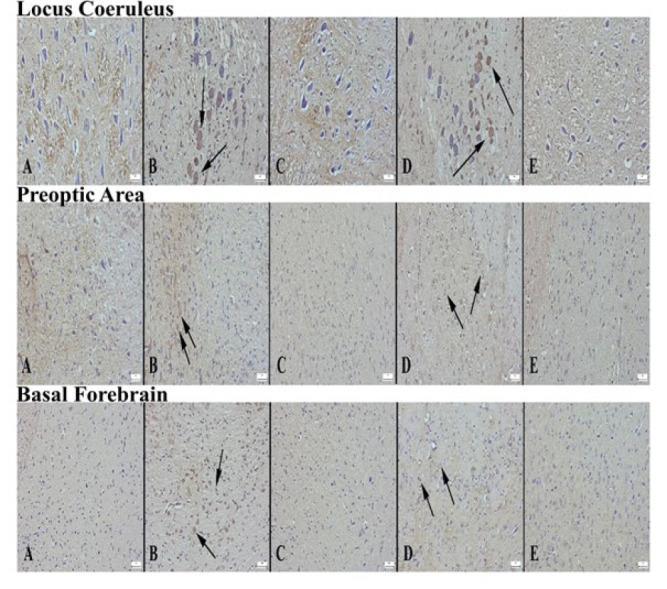
Appearance of ADRB2 expression in locus coeruleus area of the groups of rats (A) Decreased expression in the control group, (B) Significantly increased expression in the alprazolam group (arrows), (C) Increased expression in L1 neurons compared to the control group, (D) Significantly increased expression in L2 group (arrows), (E) Increased expression in L3 group neurons compared to control, Streptavidin biotin peroxidase method, Bars=50 µm. Appearance of ADRB2 expression in preoptic area according to groups (A) Decreased expression in neurons in the control group, (B) Significantly increased expression in neurons in the alprazolam group (arrows), (C) Increased expression in neurons of L1 group compared to control group (arrows), (D) markedly increased expression in L2 group (arrows), (E) increased expression in L3 group compared to control, Streptavidin biotin peroxidase method, Bars=50 µm. Appearance of ADRB2 expression in the basal forebrain area of the groups (A) Decreased expression in the control group, (B) Significantly increased expression in the alprazolam group (arrows), (C) Increased expression in L1 neurons compared to control group (arrow), (D) markedly increased expression in L2 group (arrows), (E) increased expression in L3 group compared to control, Streptavidin biotin peroxidase method, Bars=50 µm

**Figure 10:**
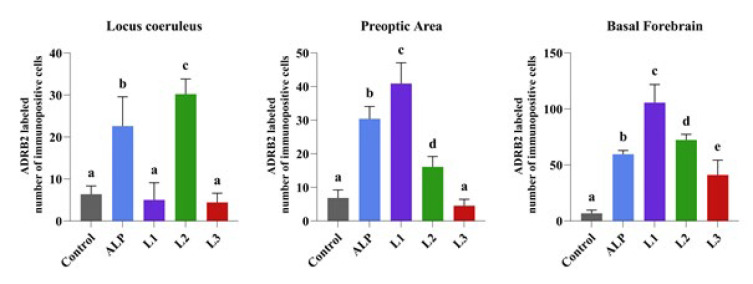
Comparison between groups of rats was evaluated using the Duncan test

Groups with the same superscript in the same column are statistically similar, and the differences between groups with different symbols indicate statistical significance (*P<*0.05). Data are presented as mean and standard deviation

## Results

***Histopathological results***

Microscopical examination of the control group’s LC, PO area, and BF tissues showed hyperemia, edema in the brain tissue, and degenerative changes in neurons. In the Alprazolam group, degenerative changes were significantly lower. Degenerative changes were lower when the L1, L2, and L3 groups were compared with the control group. Among the Lavender groups, the most significant improvement was observed in the L2 group. When ranked according to the severity of brain tissue damage, the groups were as follows: control, L3, L1, L2, and ALP. It was observed that the medicinal lavender essential oil reduced degenerative changes, although not as much as ALP. The histopathological appearance of LC, PO area, and BF between the groups is shown in Figure 2.

***Immunohistochemical results***

*c-Fos*

When LC, PO area, and BF were examined in the groups, it was seen that c-Fos expression was significantly increased in neurons in all brain areas in the control group. ALP significantly reduced c-Fos expression in LC, PO area, and BF compared to the control group. While c-Fos expression was increased in LC in the L1 group, a decrease in BF and PO area was detected. It was determined that c-Fos expression was decreased in LC, PO, and BF cells in L2 and L3 groups (Figures 3 and 4).

*ChAT*

ChAT immunoexpression in the LC, PO area, and BF was significantly decreased in the control group. Compared to the control group, ChAT immunoexpression was significantly increased in the ALP, L1, L2, and L3 groups. The most significant increase was detected in the L2 group (Figures 5 and 6). 

*GAD*

When GAD expression was examined in LC, PO area, and BF in the groups, a decrease in GAD expression was detected in the control group, while a significant increase was observed in the ALP group. In the Lavender groups, an increase in GAD expression was detected in the BF and PO region in the L1 group compared to the control group. In contrast, a significant increase was detected in the LC, BF, and PO area in the L2 and L3 groups. The most significant increase was detected in the L2 group (Figures 7 and 8).

*ADRB2*

Immunohistochemical analysis of ADRB2 expression in the groups showed that the expression was significantly reduced in neurons in LC, PO region, and BF in the control group. With ALP treatment, ADRB2 expression significantly increased in all brain regions compared to the control group. A significant increase in ADRB2 expression was detected in BF and PO region in the L1 group, in all brain regions in the L2 group, and in BF in the L3 group (Figures 9 and 10).

## Discussion

This study showed that Lavender EO inhalation improved ChAT, GAD, and ADRB2 expressions in LC, PO area, and BF in rats subjected to TSD. Improvement in ChAT, GAD, and ADRB2 expressions in LC, PO area, and BF was detected in the animals receiving Alprazolam, a standard anxiolytic drug. 

The major feature of fos protein is that its increased level indicates cell activation. Gene c-Fos is activated in neuronal cell nuclei in response to the influence of various stimuli. Therefore, the expression of fos proteins in neurons in the central nervous system is considered a marker of neuronal activity. In this manner, mapping can be performed to determine the neurons within the central nervous system that are active and those that are not firing, namely, static **(23)**. In one study, researchers used c-Fos mapping through the fluorescence micro-optical sectioning tomography (fMOST) technique to comprehensively analyze the activation state of c-Fos accumulated across the whole brain in a mouse model of chronic sleep deprivation. As a result, they detected c-Fos-positive cells in 230 brain regions. In particular, the isocortex-cerebral cortex plate area, including such areas as the retrosplenial, anterior cingulate, agranular insular, and parasubiculum, was found to be the most impacted (24). In our study, a significant decrease in c-Fos expression in LC was observed in ALP, L2, and L3 groups, while a significant increase was observed in the L1 group compared to the control group. The increase in the L1 group suggests that the applied dose was not sufficient. c-Fos expressions in the PO area and BF in Lavender EO-treated groups showed an improvement close to that of the ALP-treated group.

Cholinergic neurons are widely distributed throughout the central nervous system, particularly in the basal forebrain. Cholinergic neurons play an essential role in sleep, wakefulness, learning, and memory processes **(25)**. In a rat model of REM sleep deprivation created by Khadrawy *et al*., the researchers examined oxidant and antioxidant markers and ChAT expression in brain regions following REM sleep deprivation. As a result, they revealed that REM sleep deprivation caused oxidative stress and increased cholinergic activity (9). Researchers examined ChAT expression in all brain regions of rats subjected to REM sleep deprivation. Their results revealed high levels of ChAT expression in the pons, medulla oblongata, and thalamus. (26). Adsersen *et al*. investigated the therapeutic effects of eleven Danish medicinal plants, including *L. angustifolia*, on memory dysfunction. In their study, researchers evaluated ChAT activity and demonstrated that *L. angustifolia *showed moderate inhibitory effects, reducing ChAT activity by over 15% at a concentration of 0.1 mg/ml (27). Our study found that ChAT expressions significantly increased in LC, PO area, and BF in the ALP group used as a positive control. Similarly to the ALP group, ChAT expression significantly increased in Lavender EO-treated groups. Except for the increase in c-Fos expression in the L1 group, the increase in c-Fos and ChAT expressions similar to that of the ALP group may suggest that Lavender EO improves molecular levels in non-REM sleep. 

GABA neurons induce sleep by inhibiting several groups of neurons. A study examined differential c-Fos expression in cholinergic, monoaminergic, and GABAergic cell groups of the pontomesencephalic tegmentum after REM sleep deprivation and recovery from insomnia. The researchers observed an increase in GABAergic cell groups and a decrease in serotonergic neurons during the relief period of REM insomnia (28). Alnamer *et al*. investigated the sedative and hypnotic activities of the methanolic and aqueous extracts of *L. angustifolia *on the central nervous system. They compared the effects at 200, 400, 600, 800, and 1000 mg/kg doses with the group given Diazepam. As a result, they observed a significant sedative effect at doses of 200, 400, and 600 mg/kg and a hypnotic effect at doses of 800 and 1000 mg/kg. This study has shown that the methanolic and aqueous extracts of *L. angustifolia* have potent sedative and hypnotic effects, supporting its therapeutic use for insomnia (29). In our study, GAD expression in LC, PO area, and BF significantly increased in the ALP group. A significant increase was also detected in the groups treated with Lavender EO (except LC in the L1 group). In the L1 group, the findings regarding c-Fos and GAD expressions in LC were mutually supportive. When we evaluated both ChAT and GAD expressions in LC, PO area, and BF, we suggested that Lavender EO activates GABAergic neurons by inhibiting cholinergic neurons.

Norepinephrine is a key neurotransmitter in the hippocampus. It is synthesized in LC noradrenergic terminals and projects throughout other brain regions. Norepinephrine is mediated by both alpha- and beta-adrenergic receptors, with α2-adrenergic receptors comprising one of these subgroups. Noradrenergic neurons in LC are essential in regulating cortical activity and sleep-wake states in BF and PO (30). These neurons fire at the highest rates when animals are active and awake and at lower rates during both non-REM and REM periods of sleep (31). Strong evidence is presented that the LC-NE system exhibits wake-promoting actions. However, the specific LC terminal sites and postsynaptic receptors responsible for these actions are not as yet fully understood. PO and BF receive noradrenergic inputs from LC (32). Previous studies have examined whether NE promotes wakefulness within the subcortical regions. In these studies, the sleep-wake/arousal effects of noradrenergic α1-receptor agonist (phenylephrine) and a noradrenergic β-receptor agonist (isoproterenol) were investigated in anesthetized and unanesthetized groups. Ultimately, these receptors have been shown to promote animal alertness (33, 34). Additionally, prior research indicates that neurons in LC completely stop firing during REM sleep, but during phasic slow-wave sleep are characterized by sleep spindles and slow oscillations, LC neurons tend to fire in bursts (35,36). In the study of GABAergic neurons in urethane-anesthetized rats, researchers showed that a significant group of GABAergic neurons located in BF would be most active during slow-wave sleep along with cortical slow activity. In addition, they found that GABAergic neurons corresponded to a large proportion of previously identified sleep-active neurons in the region. These active GABAergic neurons may correspond to a large proportion of GAD+ neurons bearing α2-adrenergic receptors. The α2-adrenergic receptor is responsible for hyperpolarization of the membrane through the opening of potassium channels on preoptic area neurons as well as on locus coeruleus neurons (37). Our study investigated the immune expression of ADRB2 receptors in LC, PO area, and BF. ADRB2 expression in LC showed no significant difference between the control, L1, and L3 groups. However, ADRB2 expression increased significantly in the ALP and L2 groups. ADRB2 expression was not significantly different in the PO area between the control and L3 groups, while the ALP group showed a significant increase compared to L1 and L2 groups. In BF, ADRB2 expression was significantly increased in the ALP group and all Lavender EO-treated groups. This result suggests that the ADRB2 receptor may be associated with increased insomnia following SD. However, further studies are required to validate this finding. Explaining the potential role of the ADRB2 receptor in regulating the sleep/wake cycle would significantly contribute to the existing literature. 

## Conclusion

Our study demonstrates that Lavender EO affects the expressions of c-Fos, ChAT, GAD, and ADRB2 comparable to those of Alprazolam, a standard anxiolytic. It is worth mentioning that among the groups treated with Lavender EO, the L2 group exhibited effects most closely resembling those of the Alprazolam group. In this context, it is suggested that the 5 ml dose of Lavender EO administered to the L2 group may have a greater efficacy in improving the detrimental effects of SD than the doses used in other groups. However, further research is needed to explore whether Lavender EO treatment employs an underlying mechanism similar to Alprazolam. 

## Declaration

The authors declare that they have not used any AI tools or technologies to prepare the manuscript.
